# Dr. Blieding on Transfusion of the Blood of a Goat into the Veins of a Man

**Published:** 1842-11-19

**Authors:** 


*Transfusion of the Blood of a Goat into the Veins of a Man.* By Dr. Blieding.—A man, 38 years of age, was seized with an hæmoptysis, which continued so long, and was so violent, that the only means of saving his life appeared to be by supplying the loss of blood by transfusion. On the fifth day after the attack a canula was introduced into the median vein of his left arm, a syringe, previously heated, was filled with blood drawn from the jugular vein of a goat, and about five ounces were injected into the vein of the man. Immediately he complained of a feeling of oppression; but this soon afterwards went off. An attack of phlebitis came on next day, but was subdued in eight days, by means of cold applications alone. His strength from this day returned, and at the end of three months he was able to resume his usual occupation. It is remarked, as the interesting point of this case, that it proves that the injection of the blood of one animal into the veins of another is not necessarily fatal.—*Edin. Med. and Surg. Journ. From Journ. de Pharmacie*, May, 1842.

